# Knee subchondral bone perfusion and its relationship to marrow fat and trabeculation on multi-parametric MRI and micro-CT in experimental CKD

**DOI:** 10.1038/s41598-017-03059-3

**Published:** 2017-06-08

**Authors:** Chao-Ying Wang, Yu-Juei Hsu, Yi-Jen Peng, Herng-Sheng Lee, Yue-Cune Chang, Chih-Shan Chang, Shih-Wei Chiang, Yi-Chih Hsu, Ming-Huang Lin, Guo-Shu Huang

**Affiliations:** 1 Department and Graduate Institute of Biology and Anatomy, National Defense Medical Center, Taipei, Taiwan; 2 Department of Radiology, Tri-Service General Hospital, National Defense Medical Center, Taipei, Taiwan; 3 Division of Nephrology, Department of Medicine, Tri-Service General Hospital, National Defense Medical Center, Taipei, Taiwan; 4 Department of Pathology, Tri-Service General Hospital, National Defense Medical Center, Taipei, Taiwan; 5 Department of Pathology and Laboratory Medicine, Kaohsiung Veterans General Hospital, Kaohsiung, Taiwan; 6 Department of Mathematics, Tamkang University, New Taipei, Taiwan; 7 Graduate Institute of Biomedical Electronics and Bioinformatics, National Taiwan University, Taipei, Taiwan; 8 Institute of Biomedical Sciences, Academic Sinica, Taipei, Taiwan; 9 Department of Medical Research, Tri-Service General Hospital, National Defense Medical Center, Taipei, Taiwan

## Abstract

The pathogenesis of chronic kidney disease (CKD) is multifactorial. In the progression of CKD arthropathy, arteriosclerosis may alter the knee subchondral bone marrow by altering blood flow through the bone vasculature. Herein, multi-parametric MRI assessment, including dynamic contrast enhanced magnetic resonance imaging (DCE-MRI), magnetic resonance spectroscopy (MRS), MRI T2*, contrast enhanced MR angiography (CE-MRA), and micro-CT were applied in a rodent nephrectomy model to: 1) investigate the blood perfusion of subchondral bone marrow and its relationship to fat water content and trabeculation pattern in CKD and 2) demonstrate the feasibility of using multi-parametric MRI parameters as imaging biomarkers to evaluate the disease’s progression. Two groups of rats in our study underwent either 1) no intervention or 2) 5/6 nephrectomy. We found that in the CKD group, perfusion amplitude *A* and elimination constant *k*_*el*_ values were significantly decreased, and vascular permeability *k*_*ep*_ was significantly increased. MRS showed that fat fraction (FF) was significantly lower, water fraction (WF) was significantly higher in the CKD group. Micro-CT showed a significant loss of trabecular bone. Knee subchondral bone marrow perfusion deficiency in experimental CKD may be associated with decreased fat content, increased water content, and sparse trabeculation.

## Introduction

Chronic kidney disease (CKD) is a global health problem and has a high worldwide incidence and prevalence. Renal function impairment is often complicated by phosphocalcic metabolism disorders, which impact bone structural integrity and further lead to CKD mineral and bone disorders (CKD-MBD)^1^. CKD-MBD alters the systemic bone strength and increases the fracture risk to 2–4 times its rate in the general population^2^. Abnormal secretion of parathyroid hormone (PTH) results in cortical bone thinning and exacerbation of microarchitectural change in trabecular bone^3^. Deficiency in subchondral bone perfusion and decreased bone density may provoke cartilage damage via the cross-talk between subchondral bone and cartilage intrinsic to the pathogenesis of knee osteoarthritis^4, 5^. The weight-bearing knee joint plays an important role in the progression of arthropathy^6–9^. We suppose that the bone disorder in the appendicular skeleton might occur early in the progression of CKD-MBD. In the last decade, there has been renewed interest in the mechanisms underlying CKD arthropathy and the association of bone marrow abnormalities with changes in the functional and structural characteristics of the arterial tree^10, 11^. London reported that CKD and end-stage renal disease (ESRD) have significant associations with arterial pathology^12^. Laroche proposed that uremic osteoporosis may occur as a consequence of bone ischemia related to vascular disease^13^. However, few studies have addressed the impact of change in peripheral bone marrow blood flow on CKD development^14, 15^. The nature of bone-vascular cross-talk is still not well understood.

The knee subchondral bone marrow is highly vascularized and vulnerable^16^. Animal studies have illustrated that injury of the subchondral bone marrow can lead to cartilage damage^17–19^. CKD has also been recognized as a risk factor for osteoporosis and fracture in humans^20^. Moreover, bone marrow adipose tissue might play a role in the pathogenesis of osteoporosis^21^. Although osteoporosis is not regarded as a vascular disease, several studies have demonstrated a possible linkage between atherosclerosis and osteoporosis^22–24^. Griffith *et al*. investigated the proximal femur from 120 age-matched females by DCE-MRI, MRS, and bone mineral density (BMD) testing. They found decreased femur perfusion and increased marrow fat in osteoporotic subjects as compared to osteopenic and normal subjects^25^. Furthermore, decreased vascularization parameters and increased bone marrow fat content in vertebral bodies were associated with decreased bone density in osteoporotic patients and animal models of osteoporosis^26, 27^. Researchers also found site-specific variations in bone marrow fat quantity and in the relationships between marrow fat and bone volume^28^. Nevertheless, the knee subchondral bone marrow is undiscovered territory in the land of CKD.

MRI is an ideal *in-vivo* non-invasively imaging technique for investigating the properties of bone marrow. CE-MRA can provide excellent and high resolution morphological coverage of the peripheral vascular anatomy in a short acquisition time^29, 30^. Multi-parametric MRI enables investigation of the changes in subchondral bone marrow parameters, including fat-water content and blood perfusion. Understanding these changes may provide insight into the pathogenesis of CKD. Although the imaging characteristics of CKD-MBD on BMD testing, high-resolution peripheral quantitative computed tomography (HR-pQCT), and micro-computed tomography (μCT) have been well documented^31, 32^, the quantitative MRI evidence for change in knee subchondral bone marrow characteristics as CKD progresses is scarce. DCE-MRI provides a valuable approach to measure bone vascularization, hemodynamics, and crucial perfusion parameters^25, 26^. Previous DCE-MRI studies have successfully shown significant perfusion change in the bone marrow of lumbar vertebrae and hip joints^33, 34^. However, no study has addressed perfusion abnormalities in subchondral bone marrow in CKD. The Brix model is one of the most common two-compartment models^35^. It uses a mono-exponential decay function to describe the kinetic flow of contrast medium between the plasma and interstitial space. After Brix model fitting, three perfusion parameters can be evaluated (i.e., amplitude *A*, the exchange rate constant from the extravascular extracellular space [EES] to plasma *k*_*ep*_, and the elimination constant of the contrast medium from the plasma *k*_*el*_). Through pharmacokinetic modeling of DCE-MRI data, subchondral bone marrow microcirculation can be quantitatively investigated in CKD.

Proton MRS, because it can separate the bulk MR signal into its lipid and water signal components, is useful for evaluating the chemical metabolites of bone marrow *in vivo* ^27^. Measurement of bone marrow fat is a useful approach for diagnosing bone fragility. Li *et al*. successfully used MRS analysis of bone marrow to show that adipogenesis occurs in synchrony with the deterioration of trabecular microarchitecture^36^. MRS may be a valuable method to elucidate pathophysiological changes in osteoporotic bone marrow as CKD progresses.

Microarchitectural deterioration of osteoporotic bone in patients with CKD increases their risk of bone fractures. μCT has been successfully used in studies of trabecular bone loss or osteoporosis in animal models^37, 38^. In our study, the correlation between multiple MR functional parameters and μCT results was investigated to verify the phenomenon of trabecular bone change in CKD.

MRI T2* mapping has proven to be clinically useful because of its relative noninvasiveness and faster image acquisition time. Similar to T2 mapping, MRI T2* mapping is commonly used to discern changes in the collagenous extracellular matrix of cartilage (including collagen structure and hydration status) with higher resolution^39^. Bining HJ *et al*. found that MRI T2* mapping could also detect edematous change in subchondral bone marrow, especially at the osteochondral junction^40^.

**Figure 1:**
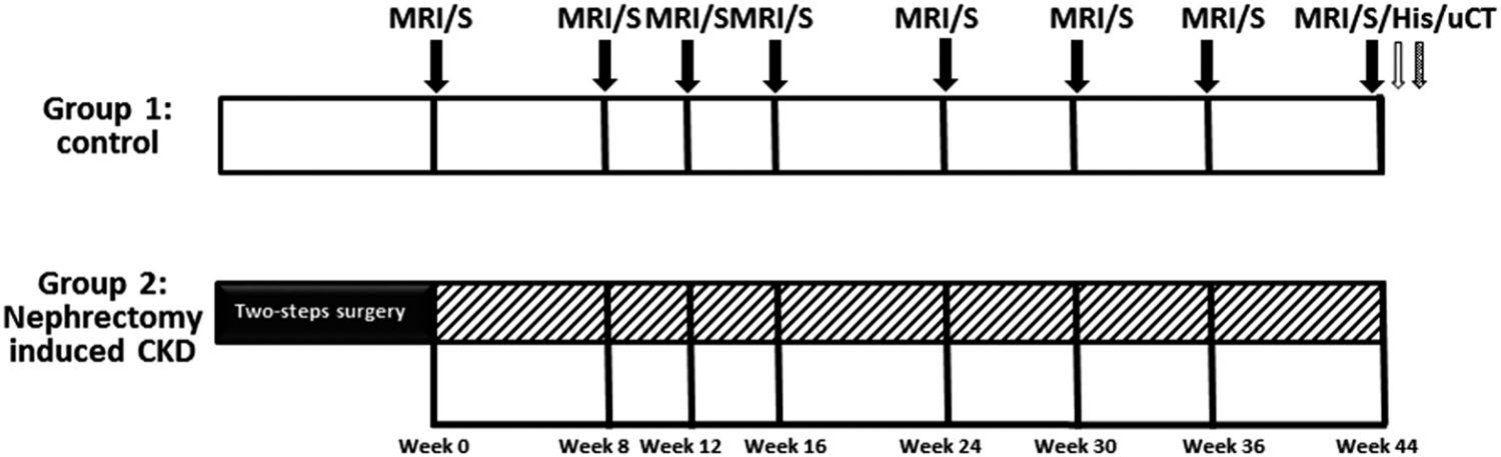
A schematic diagram of the experimental design. The chart shows the time points of MRI and MRS studies (indicated by ↓); histologic examination (indicated by ⇩), and μCT examination (indicated by ).

Knowledge of the pathophysiological processes that underlie alteration of subchondral bone marrow perfusion and its related changes in CKD can be advanced by obtaining a more detailed understanding of perfusion kinetics beyond the bone-vascular axis. We hypothesized that impaired blood supply to knee subchondral bone marrow in CKD might be a factor affecting subchondral bone marrow change. Therefore, we proposed a longitudinal multi-parametric MRI and μCT protocol to investigate changes in knee subchondral bone marrow parameters in experimental CKD, including perfusion by DCE-MRI and Brix model analysis, fat-water content by MRS, degenerative change by MRI T2*, vessel size by CE-MRA, and trabeculation by μCT (as in Fig. 1). The purposes of this study were 1) to investigate longitudinally the changes in, and the relationship between, subchondral bone marrow blood perfusion, fat water content, and trabeculation pattern in a rodent 5/6 nephrectomy model, and 2) to demonstrate the feasibility of multi-parametric MRI as an imaging modality for monitoring biomarkers of disease progression. As far as we know, this is the first work to demonstrate that longitudinal multi-parametric MRI can be used to detect and monitor knee subchondral bone marrow changes in experimental CKD.

## Results

No adverse event occurred in either experimental group. The inter-observer correlation coefficient for the regions of interest (ROIs) measurements was high (r = 0.976, 95% confidence interval [CI]: 0.962, 0.990) in all rats. The reproducibility expressed as root-mean-square coefficient of variation (CV_RMS_) was also good in all rats (9.88%; 95% CI: 8.85%, 10.91%). The mean value and standard deviation (SD) of the DCE-MRI, MRS, and MRI T2* values for the femoral and tibial knee subchondral bone marrows are shown in Table 1.

**Table 1:** The mean and SD of multi-parametric MRI parameters for the femoral and tibial subchondral bone marrow.

			Week 0	Week 8	Week 12	Week 16	Week 24	Week 30	Week 36	Week 44
			Mean ± SD	Mean ± SD	Mean ± SD	Mean ± SD	Mean ± SD	Mean ± SD	Mean ± SD	Mean ± SD
Control	Femoral	*A*	0.543 ± 0.054	0.543 ± 0.057	0.537 ± 0.054	0.504 ± 0.082	0.529 ± 0.069	0.527 ± 0.073	0.499 ± 0.062	0.474 ± 0.057
		*k*_*el*_	0.436 ± 0.027	0.432 ± 0.020	0.434 ± 0.019	0.437 ± 0.017	0.429 ± 0.025	0.422 ± 0.038	0.397 ± 0.033	0.398 ± 0.037
		*k*_*ep*_	7.425 ± 0.591	7.627 ± 0.470	7.810 ± 0.474	7.737 ± 0.550	7.945 ± 0.617	8.168 ± 0.326	7.913 ± 0.873	7.795 ± 0.417
		FF	0.134 ± 0.056	0.484 ± 0.077	0.528 ± 0.039	0.534 ± 0.046	0.532 ± 0.046	0.553 ± 0.060	0.619 ± 0.075	0.575 ± 0.091
		WF	0.866 ± 0.055	0.516 ± 0.035	0.472 ± 0.081	0.466 ± 0.046	0.468 ± 0.064	0.447 ± 0.083	0.381 ± 0.058	0.425 ± 0.091
		T2*	11.05 ± 1.122	11.53 ± 1.361	11.88 ± 1.008	11.47 ± 1.101	11.20 ± 1.018	11.93 ± 1.643	11.75 ± 1.492	12.88 ± 1.639
	Tibial	*A*	0.465 ± 0.084	0.467 ± 0.074	0.434 ± 0.063	0.421 ± 0.082	0.435 ± 0.053	0.424 ± 0.083	0.441 ± 0.090	0.380 ± 0.061
		*k*_*el*_	0.420 ± 0.021	0.423 ± 0.042	0.418 ± 0.030	0.420 ± 0.021	0.420 ± 0.025	0.411 ± 0.026	0.392 ± 0.036	0.379 ± 0.048
		*k*_*ep*_	7.493 ± 0.970	7.580 ± 0.983	7.743 ± 0.871	7.950 ± 0.547	7.952 ± 0.548	7.948 ± 0.544	7.810 ± 0.699	7.903 ± 0.393
		FF	0.127 ± 0.056	0.421 ± 0.142	0.492 ± 0.099	0.492 ± 0.084	0.493 ± 0.126	0.546 ± 0.110	0.519 ± 0.074	0.562 ± 0.060
		WF	0.873 ± 0.044	0.579 ± 0.071	0.508 ± 0.084	0.508 ± 0.091	0.507 ± 0.081	0.454 ± 0.046	0.481 ± 0.080	0.438 ± 0.081
		T2*	10.88 ± 1.338	10.56 ± 1.337	10.94 ± 0.572	10.54 ± 1.148	11.69 ± 1.089	11.45 ± 0.862	11.69 ± 1.741	12.29 ± 1.029
CKD	Femoral	*A*	0.701 ± 0.073	0.515 ± 0.041	0.430 ± 0.064	0.397 ± 0.086	0.347 ± 0.074	0.333 ± 0.085	0.344 ± 0.069	0.261 ± 0.087
		*k*_*el*_	0.429 ± 0.046	0.416 ± 0.035	0.383 ± 0.039	0.374 ± 0.036	0.359 ± 0.034	0.362 ± 0.026	0.333 ± 0.024	0.296 ± 0.038
		*k*_*ep*_	7.698 ± 0.470	7.658 ± 0.796	8.038 ± 0.742	8.173 ± 0.547	8.200 ± 0.518	7.707 ± 0.486	9.152 ± 0.476	9.587 ± 1.081
		FF	0.109 ± 0.068	0.379 ± 0.081	0.341 ± 0.122	0.359 ± 0.086	0.355 ± 0.074	0.344 ± 0.093	0.297 ± 0.092	0.275 ± 0.086
		WF	0.891 ± 0.045	0.621 ± 0.129	0.659 ± 0.086	0.641 ± 0.085	0.645 ± 0.091	0.656 ± 0.075	0.703 ± 0.075	0.725 ± 0.060
		T2*	11.41 ± 1.185	11.16 ± 0.892	12.35 ± 1.013	13.51 ± 1.299	15.76 ± 1.802	16.39 ± 2.093	16.31 ± 1.896	16.68 ± 1.386
	Tibial	*A*	0.618 ± 0.077	0.429 ± 0.035	0.295 ± 0.082	0.264 ± 0.072	0.252 ± 0.066	0.276 ± 0.064	0.274 ± 0.045	0.204 ± 0.072
		*k*_*el*_	0.428 ± 0.033	0.402 ± 0.022	0.381 ± 0.031	0.380 ± 0.020	0.346 ± 0.040	0.345 ± 0.035	0.317 ± 0.036	0.290 ± 0.057
		*k*_*ep*_	7.442 ± 0.821	7.775 ± 0.441	8.005 ± 0.898	8.018 ± 1.009	8.280 ± 0.965	8.485 ± 0.966	8.882 ± 0.883	9.417 ± 0.865
		FF	0.104 ± 0.062	0.297 ± 0.077	0.329 ± 0.084	0.324 ± 0.085	0.315 ± 0.065	0.285 ± 0.053	0.266 ± 0.077	0.259 ± 0.087
		WF	0.896 ± 0.021	0.703 ± 0.129	0.671 ± 0.073	0.676 ± 0.116	0.685 ± 0.092	0.715 ± 0.109	0.734 ± 0.139	0.741 ± 0.136
		T2*	11.20 ± 1.359	11.15 ± 1.429	11.61 ± 1.402	13.48 ± 1.356	15.03 ± 1.578	15.12 ± 1.972	15.88 ± 1.933	16.01 ± 1.844

Abbreviations: *A*: amplitude; *k*_*el*_: elimination constant; *k*_*ep*_: permeability rate constant; FF: fat fraction; WF: water fraction; T2*: relaxation time.

### DCE-MRI Analysis

Brix model analysis of bone perfusion found less than 10% variation in the three subchondral bone marrow perfusion parameters (*A*: 2.8%, *k*_*el*_: 3.2% and *k*_*ep*_: 8.9%), which indicated the Brix model had a high goodness-of-fit to the data.

**Figure 2:**
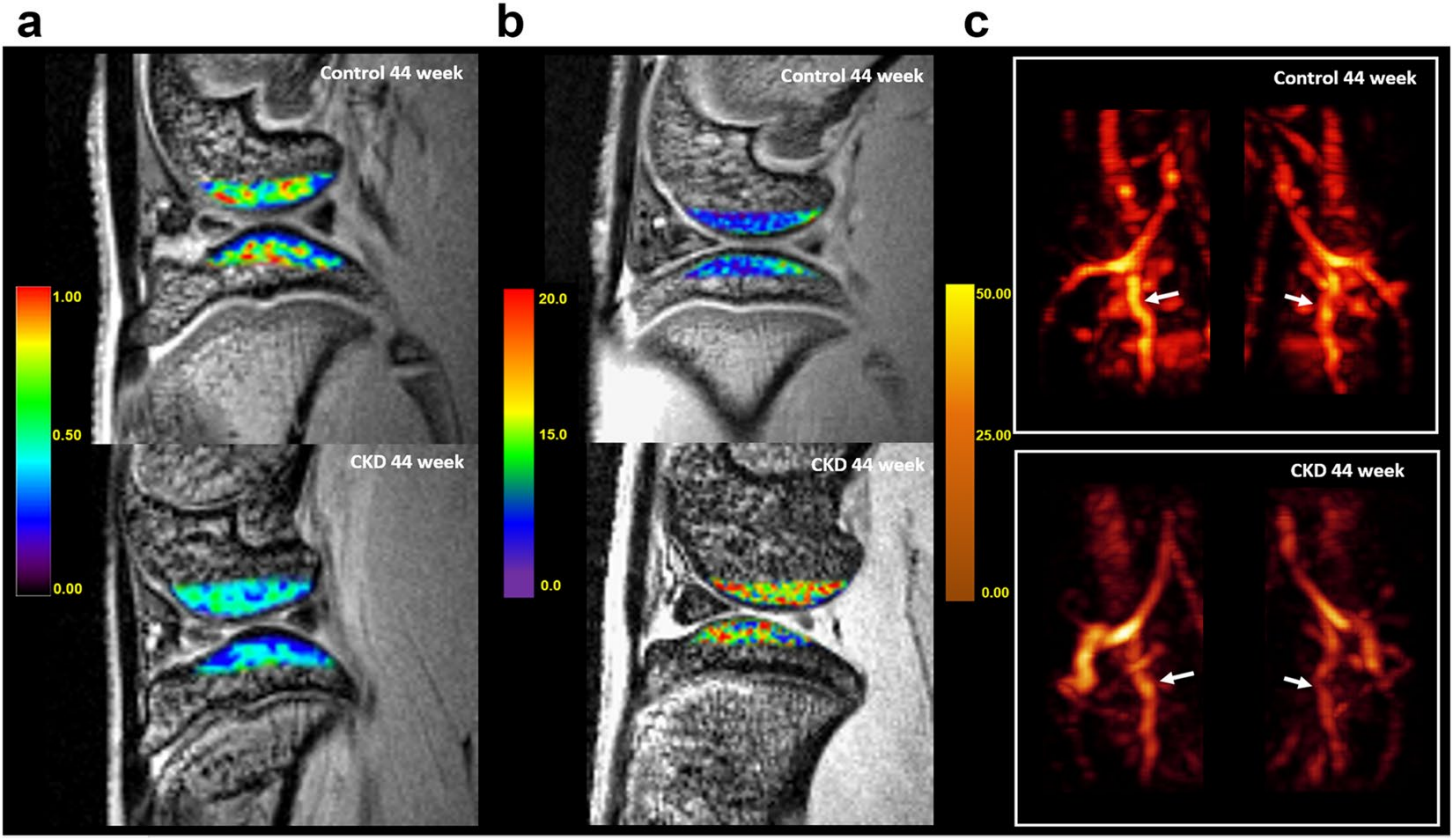
Representative DCE-MRI, MRI T2*, and CE-MRA images. Exactly 44 weeks without intervention (control rats) or after 5/6 nephrectomy (CKD rats): (**a**) DCE images (amplitude *A* map) of the lateral femoral and tibial subchondral bone marrow of the right knee show significant hypoperfusion in the CKD group (lower) but not in the control group (upper); (**b**) MRI T2* maps of the lateral femoral and tibial subchondral bone marrow of the right knee show significantly elevated T2* values in the CKD group (lower) but not in the control group (upper); and (**c**) coronal maximum intensity projections (MIPs) of the CE-MRA images of popliteal arterieheas show a significantly lower signal intensity and smaller vessel diameters in the CKD group (lower). White arrows indicate the popliteal arteries.

**Figure 3:**
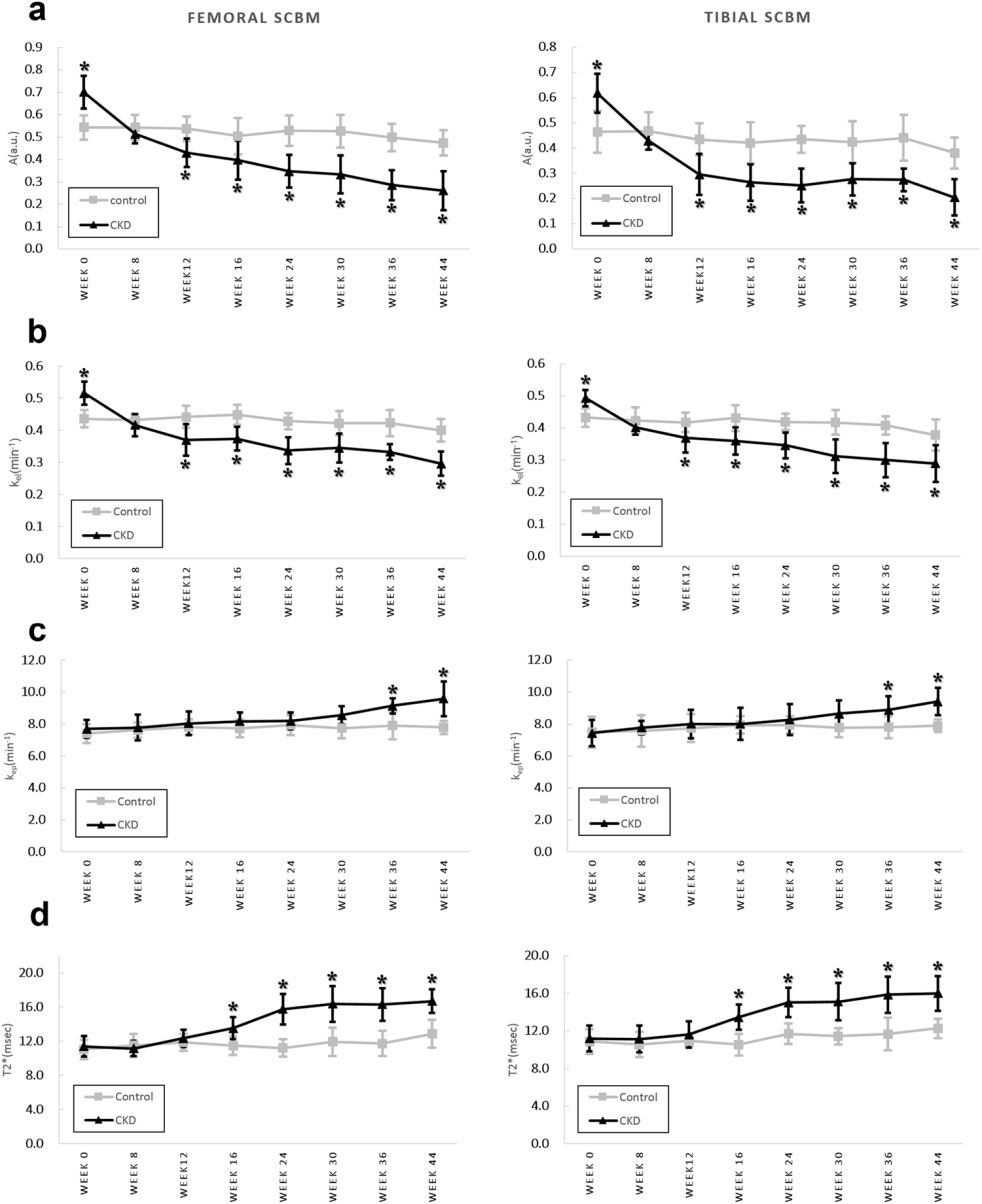
Plots showing the time courses of three perfusion parameters and the MRI T2* values (mean ± SD) for the femoral and tibial subchondral bone marrow (SCBM) of the control and CKD groups. (**a**) Amplitude *A* (unit: au). (**b**) Elimination constant *k*_*el*_ (unit: min^−1^). (**c**) Permeability rate constant *k*_*ep*_ (unit: min^−1^). (**d**) T2* (unit: msec). All values were measured in the right knees of all rats. Asterisks demonstrated significant differences (p < 0.05). A significant increase in the blood volume parameter *A* and washout parameter *k*_*el*_ can be observed in (**a**) and (**b**) at week 0, and was followed by a decrease beginning at week 8. Significant increases can be noted in the permeability rate constant *k*_*ep*_ (**c**) starting from week 36 and in MRI T2* value (i.e., edematous change) (**d**) starting from week 16.

Compared to the control group, the CKD group had significantly lower perfusion amplitude *A* and elimination constant *k*_*el*_ values in both femoral and tibial subchondral bone marrows after week 8 (all p-values < 0.001), as shown by the interaction terms in Supplementary Tables S1 and S2 and representative ROI images in Fig. 2a. On the other hand, *k*_*ep*_ in the CKD group was statistically significantly higher starting on week 36 in femoral subchondral bone marrow and on week 44 in tibial subchondral bone marrow (p-values range from 0.028 to 0.002, Supplementary Table S3). Similar variations can be seen in Fig. 3a,b and c. In the control group, compared to the baseline, the *k*_*el*_ perfusion parameter was statistically significantly decreased at 44 weeks in both femoral and tibial samples (p-values = 0.010 and 0.012 respectively). There were no statistically significant differences in *A*, *k*_*ep*_, and *k*_*el*_ in the control group at week 8, 12, 16, 24, 30, and 36.

### MRI T2* Analysis

As shown in Fig. 3d and by the interaction terms in Supplementary Table S4, the increment in MRI T2* values for the femoral and tibial subchondral bone marrows at week 16, 24, 30, 36, and 44 was significantly higher in the CKD group (2.042, 4.562, 4.453, 4.567, and 3.797) than the control group (2.935, 3.342, 3.678, 4.190, and 3.718 units; p-values ranged from 0.002 to <0.001). Representative ROI images at week 44 are shown in Fig. 2b. In the control group, the MRI T2* values for the femoral and tibial subchondral bone marrows had statistically significantly increased from baseline by 1.835 and 1.410 units at week 44 (p-values = 0.001 and <0.001, respectively).

### Single voxel ^1^H MRS Analysis

**Figure 4:**
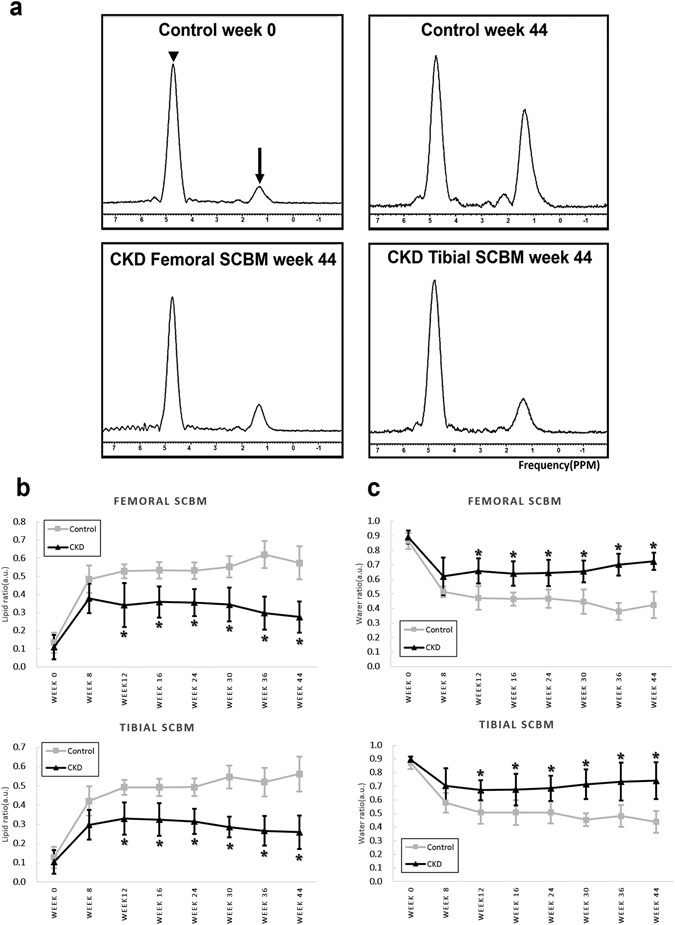
^1^H-MRS spectra of subchondral bone marrow in the control and CKD groups. (**a**) Each spectrum includes a water peak (arrow head) at 4.7 ppm and the lipid peak (arrow) at 1.33 ppm (top row, left). Spectral analysis shows that the lipid peak height increases markedly between 0 and 44 weeks in the control group (top row), but only mildly between 0 and 44 weeks in the CKD group (bottom row). (**b**) Longitudinal MRS study found that the course of change in femoral and tibial SCBM fat-water content differed between the two groups after week 8. Fat content was increased at week 8 and thereafter significantly decreased in the CKD group, while water content is significantly increased in the CKD group after week 8.

In the femoral and tibial subchondral bone marrows of the CKD group, FF values were significantly increased and peaked at week 8, thereafter decreasing gradually up to the end point (week 44), but were similar at baseline (p-value = 0.447 and 0.452, respectively). WF values were significantly decreased and bottomed out at week 8, thereafter increasing gradually up to the end point (week 44). As shown by the interaction terms of Supplementary Table S5 and Fig. 4b, FF in both femoral and tibial subchondral bone marrows was significantly lower in the CKD group than the control group from week 12 (p-values ranging from 0.019 to <0.001). WF in the CKD group was significantly higher from week 12 (p-values ranging from 0.029 to <0.001, Supplementary Table S6 and Fig. 4c). The MRS results of both femoral and tibial subchondral bone marrow at each time point are shown in Table 1; typical MRS spectrum changes are shown in Fig. 4a; and the longitudinal ^1^H-MRS plots are shown in Fig. 4b,c. In the both femoral and tibial subchondral bone marrows of the control group, the FF values were significantly increased and WF values were significantly decreased from baseline at week 8, 12, 16, 24, 30, 36, and 44 (p-values < 0.001).

### CE-MRA analysis

The average signal intensity of bilateral popliteal arteries was significantly lower in CKD group than control group (26.3 ± 4.51 a.u. and 35.8 ± 2.71 a.u., respectively, p-values < 0.05) but similar between the right and left popliteal artery. The average vessel diameters in bilateral popliteal arteries were significantly smaller in the CKD group than the control group (0.92 ± 0.08 mm vs 1.17 ± 0.02 mm; p-values < 0.05) but similar between the right and left popliteal arteries. Representative CE-MRA images at week 44 are shown in Fig. 2c.

### μCT Analysis

**Figure 5:**
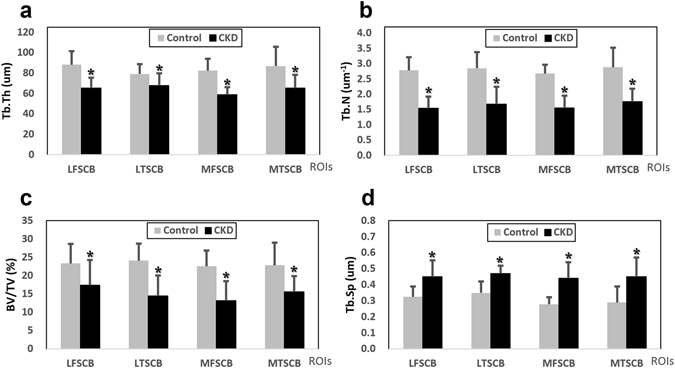
μCT analysis of the subchondral bone marrow architecture in the control and CKD groups. Four ROIs including bone marrows of the lateral femoral subchondral bone (LFSCB), lateral tibial subchondral bone (LTSCB), medial femoral subchondral bone (MFSCB), and medial tibial subchondral bone (MTSCB) were assessed and four parameters including (**a**) trabecular thickness Tb.Th (unit: µm), (**b**) trabecular number Tb.N (unit: µm^−1^), (**c**) bone volume divided by total volume BV/TV (unit: %), and (**d**) trabecular separation Tb.Sp (unit: µm) were measured at week 44 in the right knees of all rats. Subchondral bone marrows with significantly decreased Tb.Th, Tb.N, and BV/TV (**a**,**b**,**c**) and significantly increased in Tb.Sp (**d**) can be observed in the CKD group.

Compared to the control group, the CKD group had a significantly lower four-ROIs average of the following parameters: trabecular number (Tb.N) (1.63 ± 0.44 µm^−1^ vs 2.80 ± 0.47 µm^−1^; p-values < 0.018 to 0.002); trabecular thickness (Tb.Th) (64.28 ± 10.71 µm vs. 84.16 ± 13.4 µm; p-values 0.012 to <0.001); and trabecular bone volume percentage (BV/TV) (15.12 ± 5.55% vs. 23.19 ± 5.13%; p-values 0.016 to 0.007); and a higher four-ROIs average trabecular separation (Tb.Sp) (0.45 ± 0.09% vs. 0.31 ± 0.07%; p-values 0.028 to <0.001; Fig. 5a to d). Trabecular number, thickness, separation, and BV/TV were similar between the lateral, medial, femoral, and tibial ROIs of both groups (p-values > 0.05).

### Histologic Analysis

**Figure 6:**
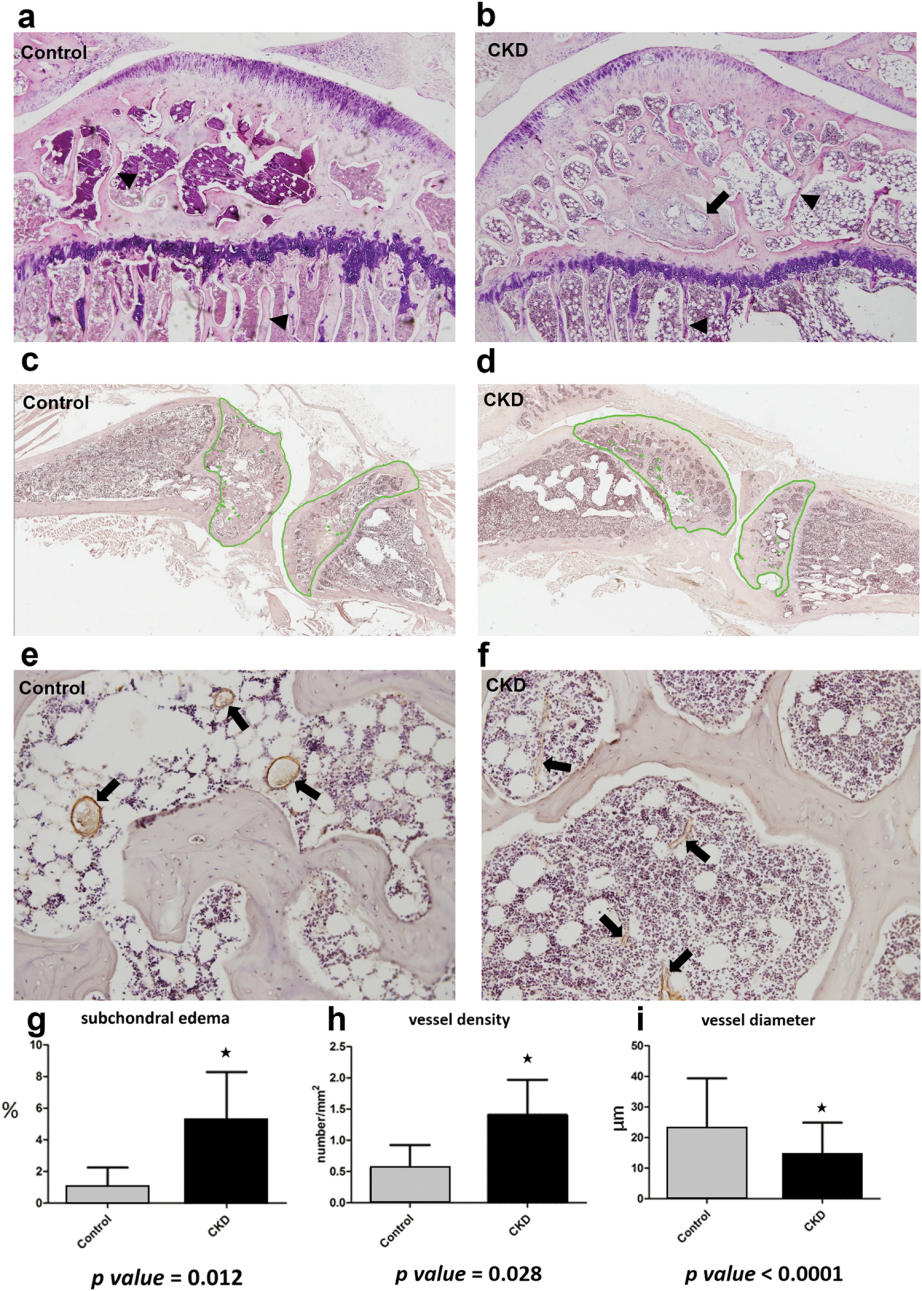
Histologic and immunohistochemical examination of the subchondral bone marrow in the control and CKD groups. Areas of subchondral bone marrow edema were statistically larger in the CKD group (**b**: arrow, H&E x40 and **g**) than in the control group (**a**: H&E x40 and **g**). Trabeculae were also thinner in the CKD group (**b**, arrowheads) than control (**a**, arrowheads). Images of subchondral marrow immunohistochemically stained with anti-alpha smooth muscle actin were analyzed using the dedicated software Image Scope (Aperio®, Leica Microsystems, Nanterre, France). The average vessel density was statistically significantly higher in CKD rats (**d**: green spots and **h**) than in control rats (**c**: green spots and **h**) and the average vessel diameter was statistically significantly smaller in CKD rats (**f**: arrows, x200 and **i**) than in control rats (**e**: arrows, x200 and **i**).

Histopathology revealed focal edematous change (arrow in Fig. 6b) in subchondral bone and thinner trabecular thickness (arrowheads in Fig. 6a and b) in the CKD group. As compared to the control group, the CKD group demonstrated statistically significantly enhanced edematous change in the subchondral bone marrow (edema area/total area: 5.25 ± 3.05% vs. 0.97 ± 1.29%; p-value = 0.012, Fig. 6g); non-statistically significant decrease in subchondral fat tissue-to-bone marrow area ratio (45.3 ± 15.3% vs. 57.0 ± 18.1%; p-value > 0.05); thinner trabecular thickness (Fig. 6a and b); statistically higher blood vessel density (1.41 ± 0.56 vessels/mm^2^ vs. 0.55 ± 0.37 vessels/mm^2^; p-value = 0.028, Fig. 6h) (as determined by anti-alpha smooth muscle immunohistochemical staining; green spots in Fig. 6c and d); and statistically smaller average vascular diameter (14.46 ± 10.44 μm vs. 22.86 ± 16.53 μm [arrows in Fig. 6e and f]; p-value < 0.0001, Fig. 6i).

## Discussion

This study demonstrates the relationship between hypoperfusion and subchondral bone marrow changes in experimental CKD. We found a decrease in subchondral bone marrow fat content and osteoporotic change of marrow trabeculation during disease progression after 5/6 nephrectomy. Moreover, to elucidate the multifactorial pathogenesis of CKD, we successfully demonstrated the feasibility of using multi-parametric MRI to track sequential changes of blood perfusion, fat-water content, and trabeculation pattern in the knee subchondral bone marrow.

We found that the DCE-MRI parameters (amplitude *A* and elimination constant *k*_*el*_) had decreased statistically significantly in both subchondral bone marrows (tibial and femoral) after 5/6 nephrectomy. Amplitude *A* is a non-specific perfusion parameter affected by vascular inflow resistance, vessel density, intraosseous pressure, and volume of interstitial space^35^. In CKD rats, the decrease in *A* may reflect changes resulting in decreased vascular inflow, especially atherosclerosis-related reduction in inflow. Although no information about the genicular artery and other upstream vessels is available, microvessels (evidence of the atherosclerotic response of subchondral bone marrow to CKD progression) can be detected by CE-MRA and histopathological examination. The elimination constant *k*_*el*_ is a systemic parameter, and prolonged perfusion time is associated with venous outflow obstruction, stasis, and decreased perfusion. Impaired vascular function and venous thromboembolism are systemic risk factors of CKD, thus we presume that decreasing *k*_*el*_ may reflect decreased outflow or the venous obstruction that develops as CKD progresses. The exchange rate constant from the EES to plasma *k*_*ep*_ is equal to the vessel permeability surface area product per unit volume of tissue relative to the size of the interstitial space, capillary endothelial permeability, and interstitial or intraosseous pressure. In our study, *k*_*ep*_ was statistically significantly higher starting from week 36. But the permeability surface area per unit volume of vasculature *Ak*_*ep*_ was decreased. Therefore, we postulate that the increase in *k*_*ep*_ may be caused by an increase in interstitial or intraosseous pressure due to the development of subchondral bone marrow edema, thereby impairing inflow and reducing the permeability of the vasculature. Moreover, the progression of uremic arteriolopathy is affected by multiple factors such as age, obesity, uremic toxins, diabetes mellitus, etc^41^. Popliteal arteriolopathy might result in hypoperfusion in the subchondral bone marrow, which then induces the proliferation of small vessels. This animal study found a correlation between microstructural change and functional abnormalities of the subchondral bone marrow vasculature.

MRI T2* relaxation time is sensitive to interactions between water molecules, extracellular matrix, and macromolecules^39^. The change in T2* or T2 values in subchondral bone marrow can be observed in conjunction with trauma, chronic cartilage damage, and osteoarthritis (OA), as an idiopathic entity or as a concomitant feature of other pathologies such as osteonecrosis or inflammation^42^. In our study, the increase in MRI T2* and water fraction (WF) values of the CKD-affected subchondral bone marrow may be related to bone marrow edema due to degeneration, or to the inflammation caused by excess fluid. The increase in subchondral bone marrow MRI T2* values due to edema began at week 16 of disease induction and was related to the decrease in marrow blood perfusion (which began at week 8). Histologic examination confirmed the presence of degenerated subchondral bone marrow (as indicated by a statistically significant increase in bone marrow edematous change). Subchondral bone marrow edema or lesions are a recognized hallmark of knee OA on MRI and regularly observed in conjunction with changes in the adjacent cartilage^43, 44^.

Regarding the μCT results, significant trabecular bone loss was found, suggesting CKD-related osteoporotic change. Osteoporosis is associated with multiple factors such as aging, obesity, atherosclerosis, and menopause. In this study, we observed atherosclerotic changes in the popliteal artery and reduced thickness and diameter of subchondral bone marrow vessels. Moreover, MRS showed a significantly lower fat content in the CKD than in the control group. Though, histopathologically, the decrease in subchondral fat tissue relative to the bone marrow area in the CKD group was not significant, the decrease in fat content was observable. These results were contrary to previously published findings on osteoporosis^36, 45, 46^. Most of the previous investigations were conducted in menopausal women or ovariectomized animals, and MRS found an increase in the fat component in osteoporosis^36, 45, 47^. However, recent studies have shown that bone-induction factors will hinder adipogenesis^48^. An *in-vitro* study demonstrated that low-concentration uric acid promotes human bone mesenchymal stem cell proliferation and osteogenic differentiation while inhibiting their adipogenic differentiation^49^. Additionally, ^1^H NMR spectroscopy showed decreased levels of lipid in 5/6 nephrectomy^50^. Although Moorthi *et al*. reported that CKD patients had significantly higher levels of vertebral bone marrow fat on MRS than healthy adults^51^, a longitudinal NMR serum study demonstrated decreased levels of lipid in the early stages of CKD in patients^52^, which is consistent with our MRS findings. Hence, we speculated that CKD-related uremic osteoporosis might be different, and that the decreased fat content might be related to an early stage response to renal dysfunction and CKD progression. The reduced subchondral bone marrow fat in MRS could be related to inhibited adipogenesis before the late stage of CKD. From our results and the assumption made above, we conclude that the decrease in fat content and increase in water content of the subchondral bone marrow may be integral to the mechanism of subchondral bone loss and marrow compositional changes during CKD progression.

Several limitations in our study should be addressed. All the rats were male. No sham groups were included and no CE-MRA, μCT, and histology were carried out at each of the time points. In addition, even though the pathogenesis of CKD arthropathy is multi-factorial, some factors affecting abnormal bone turnover such as PTH, amyloidosis, inflammatory cytokines, and uremic toxins were not investigated. Comparatively small in our sample size and possibly partial volume effects caused by changes in the subchondral bone compartment were also limitations of this work. Besides, the change in bone marrow fat content on MRS is more complicated in humans than in this experimental model. To simulate the fat content in CKD patients, a longer prospective study may be needed. Furthermore, because of differences in the nature of the axial and appendicular skeleton, weight-bearing by subchondral bone might differ from that by vertebrae and lead to different MRS results. Further study is needed to investigate the fat change mechanism in CKD.

In conclusion, structural and compositional changes noted on multi-parametric MRI, μCT, and histopathologic examinations in 5/6 nephrectomy rats provide evidence that the deficiency of blood perfusion through knee subchondral bone marrow is related to CKD. Multi-parametric MRI parameters may be clinically feasible imaging biomarkers for monitoring subchondral bone marrow changes related to CKD progression.

## Methods

All animal experiments were designed and executed following the NIH Guidelines for the Care and Use of Laboratory Animals. The study protocol was approved by the Committee on the Ethics of Animal Experiments of the National Defense Medical Center (IACUC-12-259). All surgery was operated under isoflurane anesthesia to minimize suffering. Twelve 8-week-old, male Sprague Dawley rats (each weighing about 300 g) were randomized into one of two groups: a control group (n = 6) or CKD group (n = 6). The rats were either not operated on (the control group) or subjected to subtotal 5/6 nephrectomy to induce CKD using a two-stage surgical procedure as previously reported^53, 54^. At different time points after nephrectomy, blood and urine samples, and MRI and MRS images of knee joints, were collected. The protocol is illustrated in Fig. 1. The sequential changes in the femoral and tibial subchondral bone marrow of the right knees of all rats were monitored by multi-parametric MRI at 0, 8, 12, 16, 24, 30, 36, and 44 weeks after CKD induction. The experiment was conducted with a quadrature surface coil, positioned in a 4.7T, 200/300 magnet equipped with a high-performance gradient insert (18 G/cm, 200 μs rise time; Oxford Instruments, Bruker, Ettlingen, Germany).

### DCE-MRI perfusion measurements and processing

DCE-MRI with a temporal resolution of 14.79 sec was performed using a fast gradient-echo (GRE) technique with the following parameters: TR/TE = 115.5/4.47 ms, number of excitation (NEX) = 1, matrix size = 128 × 128 reconstruction to 256 × 256, field of view (FOV) = 30 × 30 mm^2^, slice thickness (SLTH) = 0.65 mm, flip angle = 60°, bandwidth = 37.9 kHz and acquisition time = 19 min 43 s. Eighty dynamic images from each of the seven sections were acquired 60 seconds after a manual injection of 0.2 mmol gadobutrol (Gadovist; Berlin, Germany) per kilogram of body weight. The contrast medium was manually injected at a rate of about 0.01–0.02 mL/s. After the image acquisition and reconstruction, all images were processed by MIStar® software (Melbourne, Australia). The signal intensity–time data were normalized and converted to relative concentration-time data^55, 56^. The concentration-time data (*C*_*t*_) were then fitted by a nonlinear least-square curve fitting algorithm in Brix pharmacokinetic model^35^. Three perfusion parameters were obtained: the amplitude (*A*), permeability (exchange rate constant) from EES to plasma (*k*_*ep*_), and elimination constant (*k*_*el*_).

### MRI T2* measurement and processing

**Figure 7:**
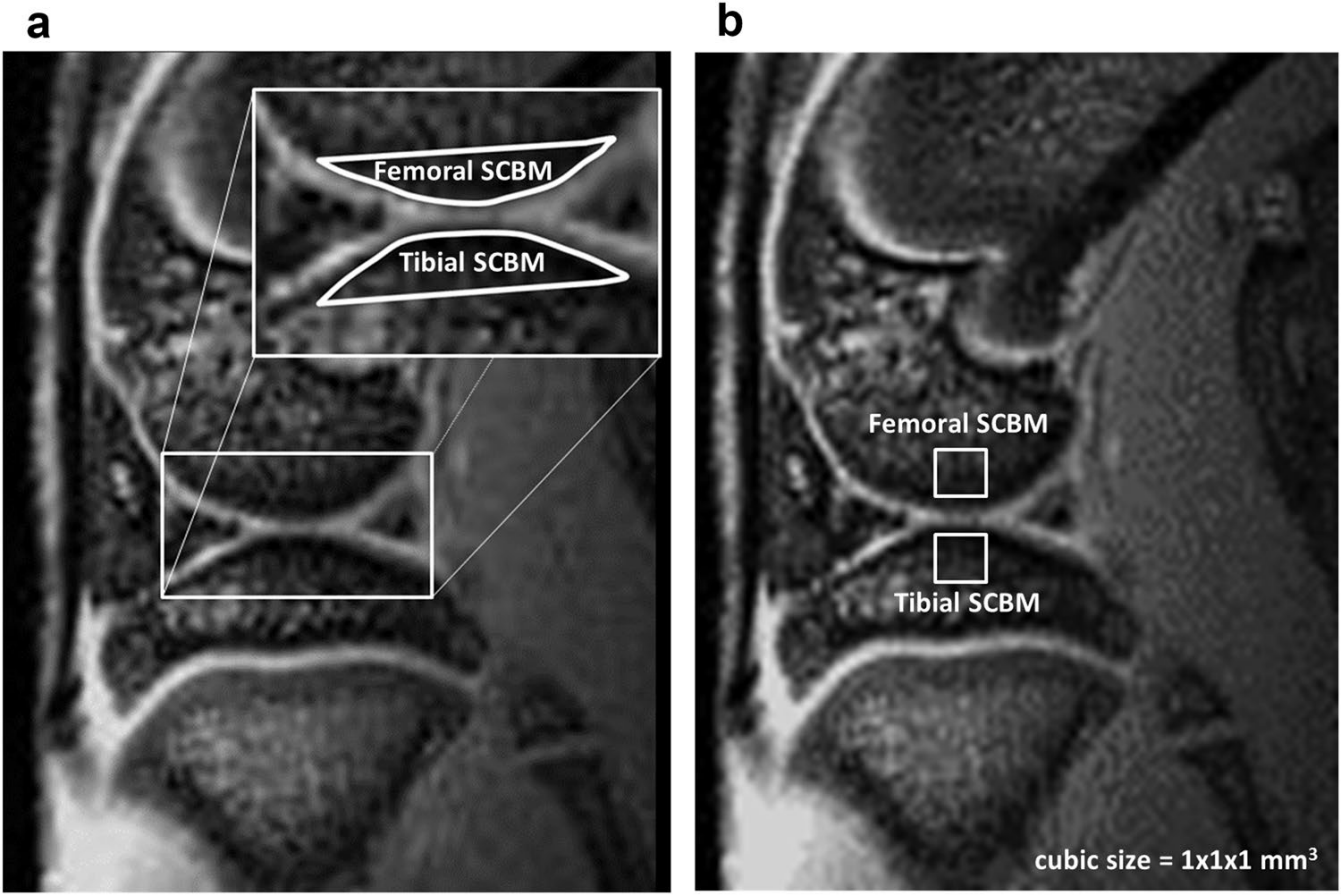
ROIs in the knee subchondral bone marrow. (**a**) ROIs (white) in the femoral and tibial subchondral bone marrow were manually selected using the DCE-MRI first-frame and MRI T2* first-echo sagittal image. (**b**) MR spectroscopy values were measured using a PRESS sequence, with two cube-shaped ROIs (1 mm each side) positioned at the center of the lateral and medial parts of the femoral and tibial subchondral bone marrow, respectively.

MRI T2* measurements were evaluated using a multiecho fast GRE sequence of seven contiguous sagittal sections without interslice gaps. The sequence had the following parameters: TR = 600 ms, 8 TEs = 3.5, 8.5, 13.5, 18.5, 23.5, 28.5, 33.5, and 38.5 ms, NEX = 12, matrix size = 256 × 192 reconstruction to 256 × 256, FOV = 30 × 30 mm^2^, SLTH = 0.65, flip angle = 30°, bandwidth = 69.4 kHz, and acquisition time = 30 min 43 s. The single exponential curve fitting method with least squares algorithm was adopted to calculate the T2* relaxation time in the selected subchondral ROIs, and to reduce the fitting errors^57^. After fitting the signal intensities from the echoes of the multiecho sequence to a monoexponential decay model, the spin density M_0_ and T2* relaxation time can be determined. The ROIs were analyzed manually on the 1^st^ echo MRI T2* sagittal image, and the ROIs of DCE-MRI were selected in 1^st^ frame images in the same manner (Fig. 7a). The average number of pixels included in both subchondral bone marrow ROIs was about 180. We selected four non-overlapping ROIs: one each for the marrows of the lateral and medial, and femoral and tibial subchondral bone at the femorotibial joint, and calculated the intensity. Averaged values for femoral and tibial subchondral bone marrow lesions were calculated from the lateral and medial ROI data.

### Single voxel ^1^H-MRS measurements and processing

MRS was acquired in 4 ROIs in marrows of the subchondral bone including the lateral and medial, femoral and tibial aspects using the Point REsolved Spectroscopy Sequence (PRESS). The parameters of PRESS were: TR/TE = 3000/35 ms, NEX = 512 without water suppression, bandwidth = 4960.3 Hz, data points = 4096, voxel size = 1.0 × 1.0 × 1.0 mm^3^, and acquisition time = 25 min 36 sec for each ROI. The PRESS boxes were positioned in the middle of the subchondral bone marrow near the osteochondral junction (as illustrated in Fig. 7b). In MRS processing, fat-water content was expressed as a percentage as previously described^58^. The percentage fat fraction of subchondral bone marrow was calculated using the large lipid peak at 1.3 ppm (saturated lipids), omit the smaller lipid peaks at 5.3 (unsaturated lipids) and 2.0 (residual lipids)^45, 46^. Fat-water content was then calculated as: *FF* = [*I*_*fat*_ */*(*I*_*fat*_ + *I*_*water*_)] × *100%*, *WF* = [*I*_*water*_ */*(*I*_*fat*_ + *I*_*water*_)] × *100%* where *I*_*fat*_ is the peak amplitude of the fat spectrum and *I*_*water*_ is the peak amplitude of the water spectrum. Averaged MRS values of both subchondral bone marrows were calculated from the lateral and medial ROIs, respectively.

### CE-MRA measurement and processing

CE-MRA of popliteal artery was performed to evaluate the knee vasculature using a fast 2D gradient-echo technique with the following parameters: TR/TE = 15.5/2.8 ms, NEX = 1, matrix size = 128 × 128 reconstruction to 256 × 256, FOV = 60 × 60 mm^2^, SLTH = 0.5 mm, flip angle = 35°, bandwidth = 68.9 kHz, and acquisition time = 5 min 45 sec. The study was acquired after a manual bolus injection of 0.2 mmol gadobutrol (Gadovist; Bayer Schering) per kilogram of body weight. A total of 15 dynamic CE-MRA images were obtained with a temporal resolution of 23 sec, resulting in a total scan time of 5 min 45 sec. The contrast medium injection was immediately followed by injection of 5 mL of normal saline solution to flush the vein. After image acquisition, we measured the signal intensity and vessel diameters of the popliteal artery. The phase of best vascular enhancement was decided subjectively and the image mask was subtracted from the unenhanced series^29, 30^. Images were evaluated using maximum intensity projections (MIP) to assess the bilateral popliteal signal intensity and vessel diameter changes.

### μCT assessment and processing

The knee was assessed using μCT (Bruker SkyScan1076, Ettlingen, Germany) with acquisition parameters as follows: source energy = 59 kVp, intensity = 167 μA, voxel size = 15 μm^3^, exposure time = 460 ms per frame, the rotation step = 0.5°, with a complete rotation over 360°, and total data acquisition time = 7 min 16 sec. Trabecular bone was analyzed at the lateral and medial, femoral and tibial subchondral bone marrows. Trabecular regions of subchondral bone marrow were selected in each two-dimensional transverse slice using manually drawn contours that exclude the cortical shell^36, 37^. After CKD induction, four parameters of bone mineral change including trabecular bone volume percentage (BV/TV, unit: %), trabecular number (Tb.N, unit: µm^−1^), thickness (Tb.Th, unit: µm), and separation (Tb.Sp, unit: µm) were evaluated using the manufacturer’s analysis tools.

To reduce the manual positioning discrepancies of the ROIs, the interobserver variability was evaluated^59^. Two well-trained quantitative image analysts (CYW: 16 years’ experience, SWC: 6 years’ experience) drew the subchondral bone marrow ROIs independently, and the ROIs were confirmed by musculoskeletal radiologist (GSH: 25 years’ experience). All results were the mean of two measurements^60, 61^.

### Histologic and Immunohistochemical Analysis

At week 44, all rats were sacrificed and their right knee joints were removed. The samples were fixed in 10% buffered formalin for 12 hours, and then decalcified in a fast decalcifier, a solution of ethylene-diamine-tetra-acetic acid. The samples were cut in half through the midsagittal line, embedded in paraffin, and cut into 3-µm thick histological sections after decalcification. The sections were stained with hematoxylin and eosin (HE), stained with anti-alpha smooth muscle actin (1:400, ab7817, Abcam, USA) using a Dako EnVision system (Dako, Carpinteria, CA) to visualize the vessels in the subchondral bone marrow, and imaged using the AT Turbo scanner (Aperio®, Nanterre, France) to evaluate the number and diameter of the vessels and to determine the subchondral bone marrow edema percentage (i.e., edema area divided by total bone area [unit: %]). Histological analysis was performed to determine the extent of fatty change (i.e., fat tissue area divided by bone marrow tissue area ×100 [%]) and vessel density in the subchondral bone (i.e., vessel number divided by subchondral bone area [number/mm^2^]).

### Statistical Analysis

The mean values and SD for the femoral and tibial subchondral bone marrow, and μCT and CE-MRA values were calculated for each group. The generalized estimating equations (GEE) method’s multiple linear regression model was used to compare intergroup differences at different time points, with groups (CKD and control), time (0, 8, 12, 16, 24, 30, 36, and 44 weeks), and their interaction terms included in the model^62^. All results were analyzed by using SPSS v22.0 software. The working correlation matrix in the GEE method was specified as autoregressive of order 1, AR(1). The interobserver agreement was calculated by using the intraclass correlation coefficient (ICC). The CV_RMS_ was used to assess the reproducibility of the analysis. All hypothesis tests were two sided and conducted at the 0.05 alpha levels.

## Electronic supplementary material

Supplementary tables
