# Supplementary material for: Knee subchondral bone perfusion and its relationship to marrow fat and trabeculation on multi-parametric MRI and micro-CT in experimental CKD
Source: Sci Rep. 2017 Jun 8;7:3073. doi: 10.1038/s41598-017-03059-3 (PMC5465086; doi:10.1038/s41598-017-03059-3)
Supplement: Supplementary file 1 — Supplementary tables [file 41598_2017_3059_MOESM1_ESM.pdf]

Supplementary Information

Knee subchondral bone perfusion and its relationship to marrow fat and trabeculation on multi-parametric MRI and micro-CT in experimental CKD

Chao-Ying Wang[1,2], Yu-Juei Hsu[3], Yi-Jen Peng[4], Herng-Sheng Lee[4,5],

Yue-Cune Chang[6], Chih-Shan Chang[2,4], Shih-Wei Chiang[7,2], Yi-Chih Hsu[2],

Ming-Huang Lin[8], Guo-Shu Huang[2,9*]

[1]Department and Graduate Institute of Biology and Anatomy, National Defense Medical Center, Taipei, Taiwan

[2]Department of Radiology, Tri-Service General Hospital, National Defense Medical Center, Taipei, Taiwan

[3]Division of Nephrology, Department of Medicine, Tri-Service General Hospital, National Defense Medical Center, Taipei, Taiwan

[4]Department of Pathology, Tri-Service General Hospital, National Defense Medical Center, Taipei, Taiwan

[5]Department of Pathology and Laboratory Medicine, Kaohsiung Veterans General Hospital, Kaohsiung, Taiwan

[6]Department of Mathematics, Tamkang University, New Taipei City, Taiwan

[7]Graduate Institute of Biomedical Electronics and Bioinformatics, National Taiwan

University, Taipei, Taiwan

[8]Institute of Biomedical Sciences, Academic Sinica, Taipei, Taiwan

[9]Department of Medical Research, Tri-Service General Hospital, National Defense Medical Center, Taipei, Taiwan

[*]Correspondence to:

Guo-Shu Huang, M.D.,

Professor of Radiology, Chief of Musculoskeletal Imaging,

Department of Radiology, Tri-Service General Hospital,

National Defense Medical Center,

325, Sec 2, Cheng-Kung Road, Neihu 114, Taipei, Taiwan, R.O.C.

Tel: (886-2)8792-7244

Fax: (886-2)8792-7245 E-mail: gsh5@seed.net.tw

Submit to *Scientific Reports* as an original research article

Supplementary Table S1 Comparisons of the femoral and tibial perfusion parameter *A* in the control and CKD groups over 44 weeks using GEE method's multiple linear regression

		Femoral				Tibial			
	Parameters	B	Std error	95% Wald C.I.	p-value	B	Std error	95% Wald C.I.	p-value
	Intercept	0.543	0.0200	(0.504 , 0.582)	< 0.001	0.465	0.0312	(0.403,0.526)	< 0.001
	CKD vs Control	0.158	0.0337	(0.092 , 0.224)	< 0.001	0.153	0.0424	(0.070,0.237)	< 0.001
**Change at each time point relative to week 0 in the control group**	Week 8 vs Week 0	–0.001	0.0143	(–0.029, 0.028)	0.972	0.003	0.0527	(–0.100,0.106)	0.957
	Week 12 vs Week 0	–0.006	0.0241	(–0.054, 0.041)	0.793	–0.031	0.0351	(–0.099,0.038)	0.385
	Week 16 vs Week 0	–0.039	0.0329	(–0.104, 0.025)	0.232	–0.044	0.0417	(–0.125,0.038)	0.295
	Week 24 vs Week 0	–0.014	0.0284	(–0.070, 0.041)	0.614	–0.030	0.0483	(–0.124,0.065)	0.539
	Week 30 vs Week 0	–0.016	0.0419	(–0.099, 0.066)	0.694	–0.041	0.0451	(–0.129,0.048)	0.369
	Week 36 vs Week 0	–0.044	0.0325	(–0.108, 0.019)	0.171	–0.024	0.0393	(–0.101,0.053)	0.541
	Week 44 vs Week 0	–0.069	0.0392	(–0.146 , 0.008)	0.078	–0.084	0.0461	(–0.174,0.006)	0.068
**Difference between the CKD and control groups at each time point**	Group[a] × Week 8	–0.186	0.0369	(–0.258, –0.114)	< 0.001	–0.192	0.0579	(–0.305,–0.079)	< 0.001
	Group × Week 12	–0.265	0.0360	(–0.336, –0.194)	< 0.001	–0.292	0.0648	(–0.419,–0.165)	< 0.001
	Group × Week 16	–0.265	0.0555	(–0.374, –0.156)	< 0.001	–0.311	0.0603	(–0.429,–0.192)	< 0.001
	Group × Week 24	–0.339	0.0454	(–0.428, –0.250)	< 0.001	–0.337	0.0528	(–0.440,–0.233)	< 0.001
	Group × Week 30	–0.351	0.0538	(–0.457, –0.246)	< 0.001	–0.302	0.0494	(–0.399,–0.205)	< 0.001
	Group × Week 36	–0.371	0.0432	(–0.455, –0.286)	< 0.001	–0.320	0.0553	(–0.428,–0.212)	< 0.001
	Group × Week 44	–0.371	0.0520	(–0.473, –0.269)	< 0.001	–0.330	0.0524	(–0.433,–0.227)	< 0.001

a: Group=1 is the CKD group; 0 is the control group (Reference group), B: regression coefficient ; C.I. : Confidence Interval

Supplementary Table S2 Comparisons of the femoral and tibial perfusion parameter k_{el} in the control and CKD groups over 44 weeks using GEE method's multiple linear regression

		Femoral				Tibial			
	Parameters	B	Std error	95% Wald C.I.	p-value	B	Std error	95% Wald C.I.	p-value
	Intercept	0.436	0.0101	(0.416,0.456)	< 0.001	0.433	0.0105	(0.412,0.454)	< 0.001
	CKD vs Control	0.080	0.0166	(0.047,0.113)	< 0.001	0.061	0.0141	(0.033,0.089)	< 0.001
**Change at each time point relative to week 0 in the control group**	Week 8 vs Week 0	–0.004	0.0094	(–0.022,0.015)	0.684	–0.010	0.0179	(–0.045,0.025)	0.588
	Week 12 vs Week 0	0.006	0.0142	(–0.022,0.034)	0.672	–0.015	0.0168	(–0.048,0.018)	0.366
	Week 16 vs Week 0	0.012	0.0122	(–0.011,0.036)	0.306	–0.002	0.0220	(–0.045,0.042)	0.940
	Week 24 vs Week 0	–0.007	0.0066	(–0.020,0.006)	0.291	–0.014	0.0148	(–0.042,0.015)	0.361
	Week 30 vs Week 0	–0.014	0.0137	(–0.041,0.013)	0.320	–0.015	0.0167	(–0.048,0.017)	0.358
	Week 36 vs Week 0	–0.012	0.0230	(–0.058,0.033)	0.587	–0.025	0.0158	(–0.056,0.006)	0.118
	Week 44 vs Week 0	–0.036	0.0140	(–0.063,–0.009)	0.010	–0.055	0.0217	(–0.097–,0.012)	0.012
**Difference between the CKD and control groups at each time point**	Group[a] × Week 8	–0.096	0.0122	(–0.120,–0.072)	< 0.001	–0.082	0.0206	(–0.123,–0.042)	< 0.001
	Group × Week 12	–0.152	0.0181	(–0.188,–0.116)	< 0.001	–0.110	0.0248	(–0.158,–0.061)	< 0.001
	Group × Week 16	–0.154	0.0141	(–0.182,–0.127)	< 0.001	–0.132	0.0276	(–0.186,–0.078)	< 0.001
	Group × Week 24	–0.172	0.0218	(–0.214,–0.129)	< 0.001	–0.134	0.0263	(–0.186,–0.083)	< 0.001
	Group × Week 30	–0.157	0.0293	(–0.215,–0.100)	< 0.001	–0.167	0.0292	(–0.224,–0.109)	< 0.001
	Group × Week 36	–0.171	0.0240	(–0.218,–0.123)	< 0.001	–0.169	0.0283	(–0.224,–0.113)	< 0.001
	Group × Week 44	–0.184	0.0183	(–0.220,–0.148)	< 0.001	–0.149	0.0322	(–0.213,–0.086)	< 0.001

a: Group=1 is the CKD group; 0 is the control group (Reference group), B: regression coefficient; C.I. : confidence interval

Supplementary Table S3 Comparisons of the femoral and tibial perfusion parameter k_{ep} in the control and CKD groups over 44 weeks using GEE method's multiple linear regression

		Femoral				Tibial			
	Parameters	B	Std error	95% Wald C.I.	p-value	B	Std error	95% Wald C.I.	p-value
	Intercept	7.425	0.2204	(6.993,7.857)	< 0.001	7.493	0.3617	(6.784,8.202)	< 0.001
	CKD vs Control	0.273	0.3041	(–0.323,0.869)	0.369	–0.052	0.4737	(–0.980,0.877)	0.913
**Change at each time point relative to week 0 in the control group**	Week 8 vs Week 0	0.202	0.1785	(–0.148, 0.552)	0.259	0.087	0.4252	(–0.747,0.920)	0.839
	Week 12 vs Week 0	0.385	0.1751	(0.042 , 0.728)	0.028	0.250	0.5480	(–0.824,1.324)	0.648
	Week 16 vs Week 0	0.312	0.2554	(–0.189, 0.812),	0.222	0.457	0.3624	(–0.254,1.167)	0.208
	Week 24 vs Week 0	0.520	0.3153	(–0.098, 1.138)	0.099	0.458	0.4883	(–0.499,1.415)	0.348
	Week 30 vs Week 0	0.328	0.4011	(–0.203, 0.863)	0.225	0.288	0.4742	(–0.641,1.218)	0.543
	Week 36 vs Week 0	0.488	0.3728	(–0.242, 1.219)	0.190	0.317	0.4455	(–0.557,1.190)	0.477
	Week 44 vs Week 0	0.370	0.1563	(0.064 , 0.676)	0.018	0.410	0.3332	(–0.243,1.063)	0.219
**Difference between the CKD and control groups at each time point**	Group[a] × Week 8	–0.112	0.4256	(–0.946,0.723)	0.793	0.247	0.5725	(–0.875,1.369)	0.667
	Group × Week 12	–0.045	0.3360	(–0.704,0.614)	0.893	0.313	0.7995	(–1.254,1.880)	0.695
	Group × Week 16	0.163	0.4224	(–0.665,0.991)	0.699	0.120	0.7361	(–1.323,1.563)	0.870
	Group × Week 24	–0.018	0.3781	(–0.759,0.723)	0.961	0.380	0.7348	(–1.060,1.820)	0.605
	Group × Week 30	0.530	0.5426	(–0.533,1.593)	0.329	0.922	0.6413	(–0.335,2.179)	0.151
	Group × Week 36	0.965	0.4391	(0.104,1.826)	0.028	1.123	0.6910	(–0.231,2.478)	0.104
	Group × Week 44	1.518	0.4797	(0.578,2.459)	0.002	1.565	0.5135	(0.559,2.571)	0.002

a: Group=1 is the CKD group; 0 is the control group (Reference group), B: regression coefficient; C.I. : confidence interval

Supplementary Table S4 Comparisons of the femoral and tibial MRI T2* values in the control and CKD groups over 44 weeks using GEE method's multiple linear regression

		Femoral				Tibial			
	Parameters	B	Std error	95% Wald C.I.	p-value	B	Std error	95% Wald C.I.	p-value
	Intercept	11.045	0.4180	(10.226,11.864)	< 0.001	10.880	.4988	(9.902,11.858)	< 0.001
	CKD vs Control	0.362	0.6081	(–0.830,1.553)	0.552	.318	.7107	(–1.075,1.711)	0654
**Change at each time point relative to week 0 in the control group**	Week 8 vs Week 0	0.480	0.1564	(0.174,0.786)	0.002	–0.320	0.7386	(–1.768,1.128)	0.665
	Week 12 vs Week 0	0.830	0.3199	(0.203,1.457)	0.009	0.055	0.3207	(–0.573,0.683)	0.864
	Week 16 vs Week 0	0.427	0.2730	(–0.108,0.962)	0.118	–0.340	0.3854	(–1.095,0.415)	0.378
	Week 24 vs Week 0	0.152	0.1320	(–0.107,0.410)	0.250	0.812	0.2724	(0.278,1.346)	0.003
	Week 30 vs Week 0	0.888	0.5061	(–0.104,1.880)	0.079	0.565	0.4495	(–0.316,1.446)	0.209
	Week 36 vs Week 0	0.700	0.2127	(0.283,1.117)	0.001	0.807	0.4588	(–0.093,1.706)	0.079
	Week 44 vs Week 0	1.835	0.5273	(0.801,2.869)	0.001	1.410	0.2951	(0.832,1.988)	< 0.001
**Difference between the CKD and control groups at each time point**	Group[a] × Week 8	–0.728	0.4055	(–1.523,0.066)	0.072	0.275	0.7491	(–1.193,1.743)	0.714
	Group × Week 12	0.112	0.4027	(–0.678,0.901)	0.782	0.357	0.7569	(–1.127,1.840)	0.637
	Group × Week 16	1.680	0.4729	(0.753,2.607)	< 0.001	2.617	0.8274	(0.995,4.238)	0.002
	Group × Week 24	4.200	0.8639	(2.507,5.893)	< 0.001	3.023	1.0659	(0.934,5.112)	0.005
	Group × Week 30	4.092	0.8982	(2.331,5.852	< 0.001	3.360	0.6175	(2.150,4.570)	< 0.001
	Group × Week 36	4.205	0.8750	(2.490,5.920)	< 0.001	3.872	0.5515	(2.791,4.953)	< 0.001
	Group × Week 44	3.435	0.9918	(1.491,5.379)	0.001	3.400	0.3742	(2.667,4.133)	< 0.001

a: Group=1 is the CKD group; 0 is the control group (Reference group), B: regression coefficient; C.I. : confidence interval

Supplementary Table S5 Comparisons of the femoral and tibial fat fraction in the control and CKD groups over 44 weeks using GEE method's multiple linear regression

		Femoral				Tibial			
	Parameters	B	Std error	95% Wald C.I.	p-value	B	Std error	95% Wald C.I.	p-value
	Intercept	0.134	0.0208	(0.093,0.174)	< 0.001	0.127	0.0209	(0.086,0.168)	< 0.001
	CKD vs Control	–0.025	0.0327	(–0.089,0.039)	0.447	–0.023	0.0310	(–0.084,0.037)	0.452
**Change at each time point relative to week 0 in the control group**	Week 8 vs Week 0	0.351	0.0302	(0.292,0.410)	< 0.001	0.294	0.0424	(0.211,0.377)	< 0.001
	Week 12 vs Week 0	0.395	0.0243	(0.347,0.442)	< 0.001	0.365	0.0294	(0.307,0.422)	< 0.001
	Week 16 vs Week 0	0.400	0.0288	(0.344,0.457)	< 0.001	0.365	0.0431	(0.280,0.449)	< 0.001
	Week 24 vs Week 0	0.398	0.0134	(0.372,0.424)	< 0.001	0.366	0.0385	(0.290,0.441)	< 0.001
	Week 30 vs Week 0	0.419	0.0398	(0.341,0.497)	< 0.001	0.419	0.0603	(0.301,0.537)	< 0.001
	Week 36 vs Week 0	0.486	0.0175	(0.452,0.520)	< 0.001	0.392	0.0273	(0.338,0.445)	< 0.001
	Week 44 vs Week 0	0.441	0.0506	(0.342,0.540)	< 0.001	0.434	0.0480	(0.340,0.528)	< 0.001
**Difference between the CKD and control groups at each time point**	Group[a] × Week 8	–0.081	0.0531	(–0.185,0.023)	0.127	–0.101	0.0510	(–0.201,–0.001)	0.048
	Group × Week 12	–0.162	0.0690	(–0.298,–0.027)	0.019	–0.139	0.0437	(–0.225,–0.054)	0.001
	Group × Week 16	–0.150	0.0420	(–0.233,–0.068)	< 0.001	–0.144	0.0568	(–0.256,–0.033)	0.011
	Group × Week 24	–0.152	0.0286	(–0.208,–0.096)	< 0.001	–0.155	0.0530	(–0.259,–0.051)	0.004
	Group × Week 30	–0.184	0.0620	(–0.305,–0.062)	0.003	–0.237	0.0650	(–0.365,–0.110)	< 0.001
	Group × Week 36	–0.298	0.0506	(–0.397,–0.198)	< 0.001	–0.230	0.0440	(–0.317,–0.144)	< 0.001
	Group × Week 44	–0.275	0.0620	(–0.396,–0.153)	< 0.001	–0.279	0.0601	(–0.397,–0.161)	< 0.001

a: Group=1 is the CKD group; 0 is the control group (Reference group), B: regression coefficient; C.I. : confidence interval

Supplementary Table S6 Comparisons of the femoral and tibial water fraction in the control and CKD groups over 44 weeks using GEE method's multiple linear regression

		Femoral				Tibial			
	Parameters	B	Std error	95% Wald C.I.	p-value	B	Std error	95% Wald C.I.	p-value
	Intercept	0.897	0.0204	(0.857,0.937)	< 0.001	0.911	0.0166	(0.878, 0.943)	< 0.001
	CKD vs. Control	0.011	0.0262	(–0.041,0.062)	0.688	0.039	0.0184	(0.003, 0.075)	0.033
**Change at each time point relative to week 0 in the control group**	Week 8 vs. Week 0	–0.330	0.0238	(–0.376, –0.283)	< 0.001	–0.347	0.0558	(–0.456, –0.238)	< 0.001
	Week 12 vs. Week 0	–0.397	0.0381	(–0.472, –0.323)	< 0.001	–0.343	0.0312	(–0.404, –0.282)	< 0.001
	Week 16 vs. Week 0	–0.431	0.0325	(–0.495, –0.367)	< 0.001	–0.404	0.0388	(–0.480, –0.328)	< 0.001
	Week 24 vs. Week 0	–0.407	0.0284	(–0.463, –0.351)	< 0.001	–0.389	0.0343	(–0.456, –0.322)	< 0.001
	Week 30 vs. Week 0	–0.391	0.0289	(–0.448, –0.335)	< 0.001	–0.421	0.0343	(–0.488, –0.353)	< 0.001
	Week 36 vs. Week 0	–0.449	0.0327	(–0.513, –0.385)	< 0.001	–0.413	0.0178	(–0.448, –0.378)	< 0.001
	Week 44 vs. Week 0	–0.472	0.0354	(–0.542, –0.403)	< 0.001	–0.390	0.0274	(–0.444, –0.336)	< 0.001
**Difference between the CKD and control groups at each time point**	Group[a] × Week 8	0.053	0.0383	(–0.022, 0.128)	0.170	0.046	0.0700	(–0.091, 0.183)	0.510
	Group × Week 12	0.125	0.0482	(0.031, 0.220)	0.009	0.037	0.0375	(–0.037, 0.111)	0.029
	Group × Week 16	0.200	0.0414	(0.119, 0.281)	< 0.001	0.157	0.0563	(0.047, 0.267)	0.005
	Group × Week 24	0.180	0.0397	(0.102, 0.258)	< 0.001	0.142	0.0489	(0.046, 0.238)	0.004
	Group × Week 30	0.169	0.0378	(0.094, 0.243)	< 0.001	0.196	0.0519	(0.095, 0.298)	< 0.001
	Group × Week 36	0.246	0.0460	(0.156, 0.336)	< 0.001	0.205	0.0518	(0.103, 0.306)	< 0.001
	Group × Week 44	0.287	0.0437	(0.202, 0.373)	< 0.001	0.198	0.0555	(0.090, 0.307)	< 0.001

a: Group=1 is the CKD group; 0 is the control group (Reference group), B: regression coefficient; C.I. : confidence interval
